# Zebrafish models in translational research: tipping the scales toward advancements in human health

**DOI:** 10.1242/dmm.015545

**Published:** 2014-07

**Authors:** Jennifer B. Phillips, Monte Westerfield

**Affiliations:** Institute of Neuroscience, 1254 University of Oregon, Eugene OR 97403-1254, USA.

**Keywords:** Usher syndrome, Cancer, Individualized medicine, Muscular dystrophy, Tuberculosis

## Abstract

Advances in genomics and next-generation sequencing have provided clinical researchers with unprecedented opportunities to understand the molecular basis of human genetic disorders. This abundance of information places new requirements on traditional disease models, which have the potential to be used to confirm newly identified pathogenic mutations and test the efficacy of emerging therapies. The unique attributes of zebrafish are being increasingly leveraged to create functional disease models, facilitate drug discovery, and provide critical scientific bases for the development of new clinical tools for the diagnosis and treatment of human disease. In this short review and the accompanying poster, we highlight a few illustrative examples of the applications of the zebrafish model to the study of human health and disease.

## Introduction

Since its establishment as a vertebrate model of development nearly 30 years ago, the experimental value of zebrafish has been demonstrated over a broad range of biological research. In the 1990s, large-scale mutagenesis screens (Driever et al., 1994; Mullins et al., 1994; Haffter et al., 1996) provided researchers with thousands of mutants, the phenotypic characterizations of which illuminated numerous cellular events in vertebrate development and foreshadowed the power of zebrafish as a human disease model. The 21st century ushered in the zebrafish genome project, which revealed conserved functions of myriad genes across the vertebrate lineage and identified zebrafish orthologs for 82% of the known human disease genes (Howe et al., 2013). The sequenced genome provided the community with targets for reverse genetics through widespread use of morpholinos (Nasevicius and Ekker, 2000), antisense oligonucleotides designed to temporarily downregulate gene function by blocking translation or proper splicing. Although sometimes limited by the short efficacy period and confounding off-target effects (reviewed in Bedell et al., 2011), the use of morpholinos in zebrafish research, beginning at the turn of this century, tremendously accelerated zebrafish loss-of-function studies and solidified the relevance of investigating zebrafish orthologs of human disease genes. The well-mapped genome also provided the information necessary to expand the collection of transgenic lines, which, given the optical clarity of zebrafish embryos and larvae, have enabled *in vivo* study of cellular and molecular processes in breathtaking fluorescent detail (Feng et al., 2012; Chen et al., 2013; Pan et al., 2013; Xiong et al., 2013; Staudt et al., 2014; Miesfeld and Link, 2014). Genome sequencing also revealed a whole-genome duplication event in the teleost lineage, with 25–30% of the duplicated genes having been retained in the zebrafish because of partitioning of functions that were originally performed by the single ancestral gene (subfunctionalization) (Force et al., 1999; Howe et al., 2013; Schartl, 2014; Braasch and Postlethwait, 2012). In addition to contributing to our knowledge of molecular evolution, the presence of duplicated genes with shared function in the zebrafish genome provides the novel opportunity for fine-tuned analyses of gene regulation. Most recently, the development of genome-editing techniques, including transcription activator-like effector nucleases (TALENs) (Joung and Sander, 2013) and clustered regularly interspaced short palindromic repeats (CRISPR)/Cas9 (Chang et al., 2013; Hruscha et al., 2013; Hwang et al., 2013), has enabled researchers to induce heritable mutations with remarkable precision (see poster panel 2). Finally, large-scale screens using panels of pharmaceutical compounds or small molecules have been developed over the past several years, where thousands of embryos can be allocated into multi-well plates and exposed to chemicals to test drug dose-response levels, potential for teratogenicity or toxicity, and therapeutic potential of known or novel compounds (Peterson et al., 2000; Brannen et al., 2013; Ducharme et al., 2013; Esterberg et al., 2013; Gebruers et al., 2013; Hao et al., 2013; Swartz et al., 2013; Wang et al., 2014; Westhoff et al., 2013) (see poster panel 2).

In line with the ongoing development of these methodologies, the zebrafish has become increasingly valuable as a translational research model, and is now contributing to hundreds of studies worldwide on human disease mechanisms ranging from autism (Jacob et al., 2014) to vascular disease (Fang et al., 2014). The rapid, *ex utero* development of zebrafish embryos (Kimmel et al., 1995), coupled with the speed and facility with which loss of gene function can be assayed, have been used to great advantage by researchers to conduct *in vivo* investigations of orthologs of human disease genes. Zebrafish are as accessible and proliferative as cell culture, but also provide a complex, *in vivo*, functional vertebrate model system. This unique combination of attributes is steadily gaining recognition as laboratories working on human health either in clinical settings or with other model organisms are increasingly including zebrafish studies in their comprehensive analysis of human diseases and potential treatments. This brief review and the accompanying poster summarize some of the key features of the zebrafish model system and highlight some recent examples of its utility in human disease-focused research.

## Direct impacts on human health: new tools to combat cancer and tuberculosis

Of all the stellar zebrafish research in recent years, we have chosen to highlight two fish tales with exceptional impact. The first comes from Leonard Zon and colleagues at Harvard Medical School, whose analysis of the complex genetic milieu of malignancies over the last decade has ushered in the development of powerful cutting-edge tools of the zebrafish field (see poster panel 3; example 2). In 2011, the Zon laboratory used transgenic zebrafish that express a human oncogene, *BRAF*^V600E^, under the control of a melanocyte-specific promoter (*mitfa*) in a background depleted of the well-studied tumor suppressor gene *p53* to identify chemical factors that offset aberrant regulation of neural-crest-specific genes. Dysregulation of neural-crest-specific genes is known to contribute to cancers in crest-derived tissues, such as melanocytes (tumors affecting melanocytes are termed melanoma). The group discovered that inhibitors of dihydroorotate dehydrogenase (DHODH) inhibit neural-crest development in larvae, and lead to a significant decrease in cancerous growths in the melanoma-prone zebrafish model (White et al., 2011). One such DHODH inhibitor used in the study, leflunomide, is an FDA-approved anti-arthritis drug. Based directly on the findings of White and colleagues, along with subsequent verification of DHODH-blocking activity in human and mouse cells, a Phase I/II clinical trial of leflunomide in combination with a previously studied BRAF-inhibitor was initiated and is currently underway for melanoma patients (White et al., 2013) [see also Len Zon’s account of the discovery, in this issue (Zon, 2014)].

**Figure:**
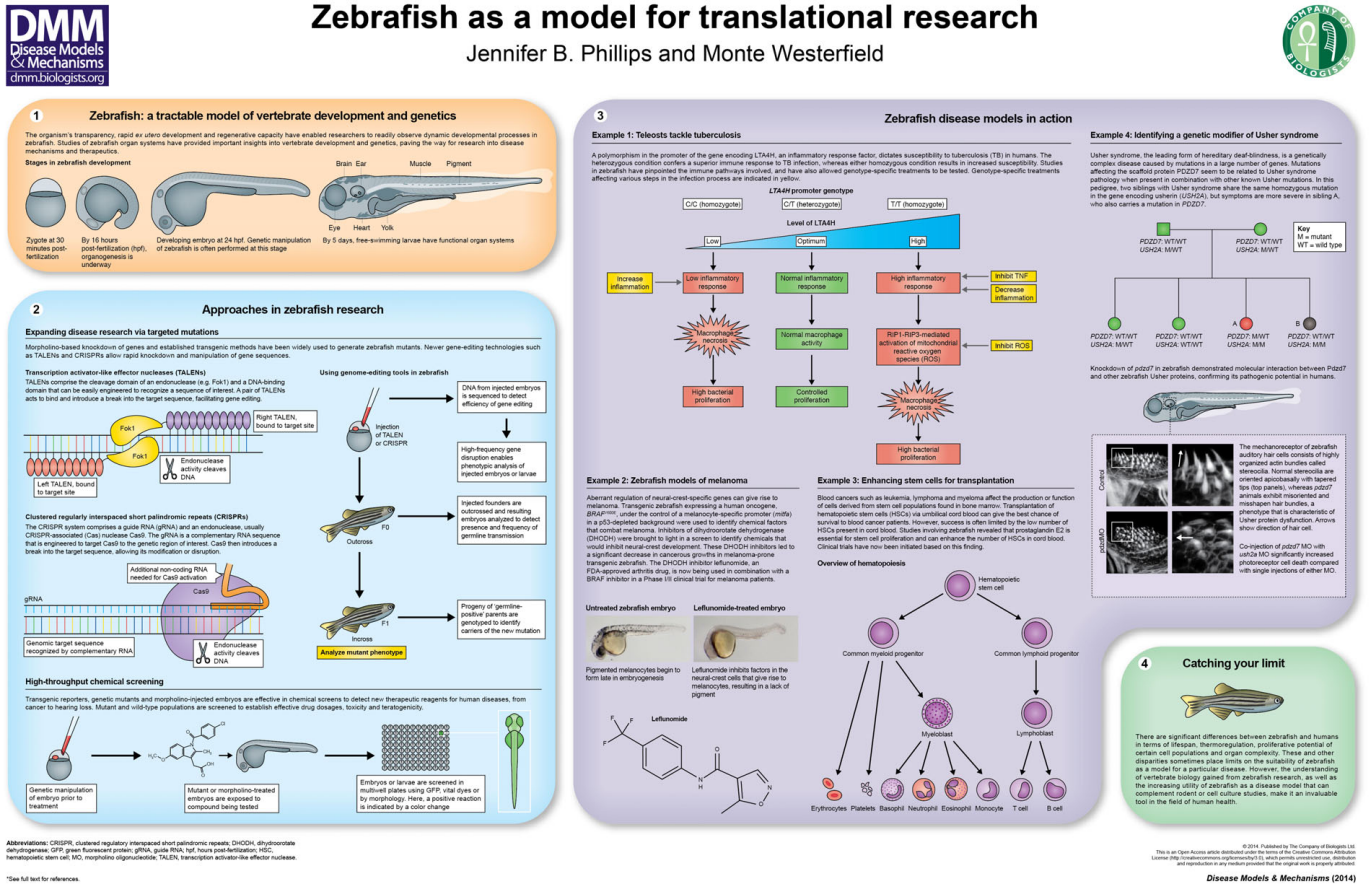


Another exemplary zebrafish success story comes from Lalita Ramakrishnan’s group at the University of Washington, who published a series of elegant papers establishing a zebrafish pathogen, *Mycobacterium marinum*, as a tractable model for the infectious disease tuberculosis (see poster panel 3; example 1). *M. marinum*, a close genetic relative of *Mycobacterium tuberculosis*, produces inflammatory granulomas in fish similar to those seen in the lung tissue of humans diagnosed with tuberculosis. Studies of the infectious process and host-immune responses of *M. marinum* can provide key insights into mycobacterial pathogenesis and provide new avenues for the treatment of human tuberculosis. Previous work by the Ramakrishnan group identified human mutations in the inflammatory response factor *LTA4H* that affect susceptibility to tuberculosis (Tobin et al., 2010; Tobin et al., 2012). Specifically, homozygous genotypes with either low or high *LTA4H* confer a more severe disease phenotype, whereas symptoms in patients with the heterozygous state are milder. Using zebrafish to recreate these genotypic combinations, Tobin and colleagues showed that human tuberculosis patients can succumb to the disease from either a hyper- or hypo-immune response. These studies provide clinicians treating tuberculosis patients with a basis for understanding variable natural history and the opportunity to provide genotype-specific treatment depending on the functional levels of LTA4H (and its downstream target, tumor necrosis factor). Building on these findings, the Ramakrishnan group has now elucidated the inflammatory response pathways that are compromised in these various patient genotypes during mycobacterial infection, implicating mitochondrial reactive oxygen species mediated by the RIP1-RIP3-dependent pathways and identifying currently available pharmaceutical compounds to target these specific response pathways in tuberculosis and other similar infections (Roca and Ramakrishnan, 2013).

## Genetic models on demand and avenues for new disease gene discovery

A PubMed search reveals an abundance of publications that characterize new zebrafish models of known human afflictions. Neurological and psychiatric disorders (Hortopan et al., 2010; Mahmood et al., 2013; Zdebik et al., 2013; Ziv et al., 2013), cancer (Ignatius et al., 2012) (reviewed in White et al., 2013), heart defects (reviewed in Staudt and Stainier, 2012; Liang et al., 2013; Staudt et al., 2014), fetal alcohol syndrome (Coffey et al., 2013; McCarthy et al., 2013; Uribe et al., 2013), ciliopathies (Austin-Tse et al., 2013; Hjeij et al., 2013) and kidney disorders (reviewed in McCampbell and Wingert, 2013; Westhoff et al., 2013) are but a few of the human diseases currently studied by zebrafish researchers. Other contributions have been made to the field of regenerative medicine, capitalizing on the regenerative capacity of zebrafish fin, retina, heart, kidney and numerous other tissues to identify the genetic factors required for replacing cells lost to injury or disease (Singh et al., 2012; reviewed in Gemberling et al., 2013; Poureetezadi and Wingert, 2013).

As a paraclinical tool, zebrafish have proven to be incredibly useful for validating the pathogenicity of newly discovered genes or alleles in human patients. Next-generation sequencing (NGS) and whole exome sequencing (WES) technologies have changed the landscape of human genetic disease diagnosis; however, distinguishing a pathogenic allele from a novel, benign polymorphism is a complex process, particularly when insufficient numbers of relatives are available for comparative sequence analysis. The ease with which potentially pathogenic genes can be depleted in zebrafish, often in combination with rescue of loss-of-function mutations by human variants to confirm their disease-causing potential, has been increasingly exploited over the past several years, offering valuable information for clinicians diagnosing Usher syndrome (Ebermann et al., 2010) (see poster panel 3; example 4), neurodegenerative diseases (Iglesias et al., 2014), muscular dystrophy (Gibbs et al., 2014), skeletal disease (Kou et al., 2013) and a broad range of other genetic disorders.

In addition to the exploration of genes that are known to impact human health, the powerful zebrafish model also contributes to the discovery of previously unidentified disease genes. For example, candidate genes obtained from large genome-wide association studies of kidney and platelet dysfunction have been screened in zebrafish to establish specific phenotypic connections to these genetic risk profiles (Pattaro et al., 2012; Gieger et al., 2011). Reverse genetics screens (Ryan et al., 2013; Huang et al., 2013; Kettleborough et al., 2013) have also shed light on new molecular partners of known disease genes, not only providing researchers with a greater understanding of the pathways involved in human disorders, but also providing new genetic targets for human geneticists to explore in patients with clinical diagnoses in the absence of a known pathogenic mutation. The potential of CRISPR/Cas9 and TALENs gene-editing techniques has been aptly demonstrated by the successful targeting of human disease genes in pioneering proof-of-concept experiments. Loss-of-function mutations in the transcription factor *GATA5* lead to severe congenital heart defects in humans (Jiang et al., 2013). CRISPR/Cas9 constructs targeting the zebrafish ortholog, *gata5*, resulted in a variety of biallelic insertions and deletions at that locus, and reproduced the heart development defects characterized in a previously identified *gata5* mutant (Chang et al., 2013). This work demonstrated the capacity of the CRISPR/Cas9 system not only to generate heritable mutations but also to induce somatic mutations in the injected animals so efficiently that a phenotype can be observed almost immediately. Now increasingly used in zebrafish research, such gene-editing techniques facilitate the rapid creation of new, custom-made mutations to investigate virtually any known or suspected disease gene. Additionally, the emerging availability of online resources such as the Human Phenotype Ontology website (http://www.human-phenotype-ontology.org/) allows cross-referenced searches for mouse and zebrafish models of human disease based on phenotypic parameters (Köhler et al., 2014).

## Netting new therapies for human disease

Beyond these indisputably valuable contributions to understanding developmental processes and the cellular and molecular bases of human diseases, numerous zebrafish models that have been generated for these purposes can now assist in finding new treatments. Chemical screens, such as the targeted method used in the Ramakrishnan *Mycobacterium* studies, can be employed on a larger scale to look for suppressors or enhancers of a given phenotype. Large numbers of developmentally synchronized, genetically homogenous zebrafish can be screened in microtiter plates, allowing for rapid evaluation of a variety of compounds and dosages in parallel. One screen for chemical effectors of hematopoiesis conducted by the Zon lab in 2007 (see poster panel 3; example 3) identified a role for prostaglandin E2 (PGE2) in stem cell regulation (North et al., 2007). As a result of this finding, along with complementary data from mouse models, clinical trials were initiated in which umbilical cord blood was treated with PGE2 prior to transplantation into leukemia or lymphoma patients (Cutler et al., 2013). The results of the Phase I trial supported the preclinical conclusion that PGE2 promotes proliferation of hematopoietic stem cells and increases engraftment, or the acceptance of transplanted cells, in the study subjects. Based on this positive outcome, a Phase II trial is now underway (Ablain and Zon, 2013).

Many other high-throughput screens using zebrafish models of ototoxicity (Esterberg et al., 2013), epilepsy (Baxendale et al., 2012; Baraban et al., 2013; Orellana-Paucar et al., 2013; Rahn et al., 2013) and cancer (Wang et al., 2010; Nguyen et al., 2012; Le et al., 2013) have uncovered new therapeutic candidates, and collectively provide a robust proof-of-principle of the extent to which zebrafish research can directly impact drug discovery.

## Summary and outlook

Throughout its history as a model organism, the zebrafish has demonstrated unique utility in embryological, developmental and genetic studies of human disease. It should be noted that some of the advantages of the zebrafish system, such as the presence of duplicated genes or the cellular regeneration potential not present in mammals, might also present limitations to the pursuit of a translational research model for a particular disease (see poster panel 4). Nonetheless, this system has much to offer the translational researcher. The expanding molecular toolkit available to the zebrafish community will continue to enhance our basic understanding of vertebrate organ, tissue, cell and molecular biological functions, and, increasingly, can be applied with ever greater precision to studies directly geared toward understanding and treating human disease. Increasing numbers of zebrafish researchers are collaborating with clinicians, and numerous scientists outside the zebrafish community are turning to zebrafish to enhance their studies as well. A comprehensive approach encompassing complementary use of multiple animal models and medically relevant technology will drive major advances in the improvement of human health in the coming years.
